# Upgrading sesame cake-extracted oil via enzymatic glycerolysis: Production and characterization of diacylglycerol-enriched oil

**DOI:** 10.1016/j.fochx.2026.104239

**Published:** 2026-07-21

**Authors:** Li Zhou, Yue Mu, Jin Zhang, Meiyu Zheng, Lei Chen, Qingyang Zhang, Jinglun Zhou, Zhengting Zhu, Shu Wang, Bin Li, Zhigang Hu, Kangyu Zhao, Dongping He, Fenfen Lei

**Affiliations:** a Key Laboratory of Edible Oil Quality and Safety for State Market Regulation, School of Food Science and Engineering, Wuhan Polytechnic University, Wuhan 430023, China; b Grain and Oil Resources Comprehensive Exploitation and Engineering Technology Research Center of State Administration of Grain, College of Food Science and Engineering, Wuhan Polytechnic University, Wuhan 430023, China; c Wuhan Institute for Food and Cosmetic Control, Wuhan 430012, China; d School of Mechanical Engineering, Wuhan Polytechnic University, Wuhan 430023, China

**Keywords:** Sesame cake solvent-extracted oil, Enzymatic glycerolysis, Diacylglycerol, Physicochemical characteristics, Lipid profile

## Abstract

Solvent-extracted oil from pressed sesame cake has limited value due to flavor loss. This study developed an enzymatic strategy to convert it into diacylglycerol (DAG)-enriched oil via Novozym 435-catalyzed glycerolysis. Under optimized conditions (molar ratio 1:4, enzyme loading 10%, 90 °C), efficient DAG formation was achieved by overcoming multiphase mass transfer limitations. The product was dominated by 1,3-DAG, with a significantly higher proportion than 1,2-DAG. Fourier transform infrared confirmed structural reconfiguration of glycerides, while gas chromatography analysis showed that polyunsaturated fatty acids were well preserved, indicating mild processing. Notably, the peroxide value of DAG oil was significantly lower than that of the original triacylglycerol oil, while the p-anisidine value remained stable, demonstrating enhanced oxidative stability. The product also exhibited a brighter yellowish-green appearance due to polar component formation. Overall, this study provides an effective pathway for upgrading sesame by-products into functional lipid ingredients.

## Highlights

• A 52.95% DAG yield was achieved from sesame oil via optimized enzymatic glycerolysis.
• Polar DAG formation was confirmed by –OH band emergence at 3476 cm⁻¹ and reduced ester C=O absorption.
• The polyunsaturated fatty acids remained stable during enzymatic glycerolysis.
• A 35.6% 1,3-DAG content was obtained, which was over two-fold higher than that of 1,2-DAG (17.4%), driven by thermodynamically favorable acyl migration.

## Graphical abstract

**Figure:**
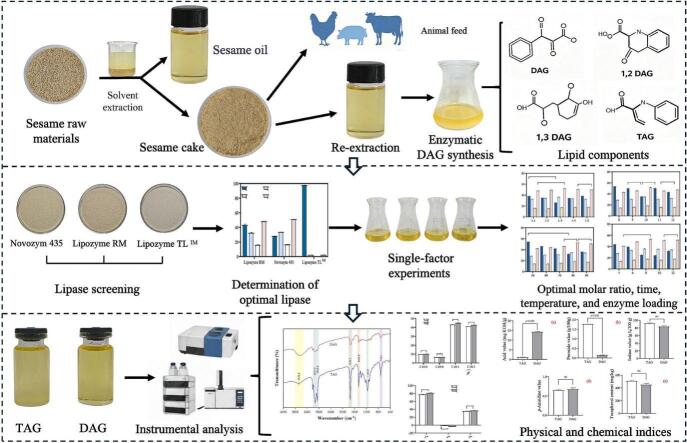

## 1 Introduction

Sesame is one of the world's most important oilseed crops, with an oil content of 44%–58% in its seeds (Matthäus & Özcan, 2018). Sesame oil is highly favored by consumers due to its rich flavor, abundant fat-soluble accompanying micronutrients, high content of unsaturated fatty acids, and excellent oxidative stability (Shi et al., 2017; Wacal et al., 2019; Zhou et al., 2026). Based on the extraction process, sesame oil is generally classified into hot-water displacement extraction sesame oil and pressed sesame oil (Zheng, Wang, et al., 2025). The principle of hot-water displacement extraction sesame oil production involves the low-temperature grinding of sesame seeds using a stone mill to disrupt the cellular structure (e.g., proteins and carbohydrates), followed by the addition of hot water via the water substitution method. This process exploits the differences in density and affinity between oil and hydrophilic substances, allowing water to displace the oil from the sesame paste, thereby achieving oil-water separation (Hwang, 2005). Pressed sesame oil is produced by physically pressing sesame seeds that have been subjected to high-temperature roasting, using mechanical force to expel the oil from the press chamber, followed by sedimentation or filtration (Piravi-Vanak et al., 2024). However, the residual oil content in sesame cake obtained after pressing remains as high as 10%–20% (Jagadeesan et al., 2023). This by-product is currently used primarily as low-value animal feed or directly discarded, resulting in a significant waste of oil and protein resources and indicating a low level of comprehensive utilization of by-products (Chaipoot et al., 2022). The solvent-extracted sesame oil obtained from pressed sesame cake using organic solvents largely loses its flavor compounds during the solvent removal process, leading to a low market value (Karataş, 2015). Therefore, identifying a high-value utilization pathway for solvent-extracted sesame oil would not only enable the resourceful utilization of this by-product.

DAG is a natural component present in low amounts in edible oils, with a molecular structure that contains one less fatty acid moiety than TAG, the major constituent of common edible oils (Hatzakis et al., 2011). In terms of physiological functions, DAG has been shown to improve lipid metabolism and reduce visceral fat accumulation, thereby exerting positive effects on the prevention and amelioration of metabolic diseases such as obesity, hyperlipidemia, and fatty liver disease (Qin et al., 2026). Its mechanism of action is primarily attributed to its unique metabolic pathway: unlike TAG, DAG is not readily resynthesized into storage fat in the body after digestion, but rather tends to enter the liver for oxidative breakdown, directly providing energy for the organism (Bakala-N'Goma et al., 2022; Shi et al., 2025). In the fields of chemical and biochemical engineering, DAG can serve as an intermediate for the synthesis of complex lipids such as structured lipids, phospholipids, and glycolipids, and has been demonstrated in enzymology studies to act as an activator or inhibitor of certain enzymes (e.g., protein kinase C) (Eichmann & Lass, 2015; Mérida et al., 2019). Currently, the preparation methods for DAG mainly include chemical and enzymatic routes (Satriana et al., 2016). The chemical method suffers from drawbacks such as cumbersome processes, high energy consumption, severe pollution, and poor product quality (Lo et al., 2008). In contrast, the enzymatic method employs lipases to catalyze the synthesis of DAG from oils and fats, offering advantages such as mild reaction conditions, controllable product structure, high conversion efficiency, and strong selectivity (Majumder et al., 2025).

Currently, research on the preparation of DAG oil has primarily focused on commodity oilseeds such as soybean oil and corn oil, with well-established optimization of process parameters and product performance evaluation (Lai et al., 2022; Lee et al., 2020). For example, using soybean oil as the substrate and Novozym 435 as the biocatalyst, Sun et al. (2020) achieved a maximum DAG yield of 49.83 ± 1.26% and a TAG conversion of 75.80 ± 0.21% via enzymatic glycerolysis in a glycerol-based deep eutectic solvent under optimal conditions (TAG/glycerol molar ratio 1:4, 80 °C, 8% enzyme load, 8 h). In parallel, studies on DAG production from specialty oils are also advancing. For instance, Wu et al. (2024) reported a maximum DAG content of 62.49% using Lipozyme RM as the biocatalyst for the enzymatic conversion of camellia seed oil via a coupled hydrolysis-esterification process. The most used enzymes for DAG production are immobilized 1,3-position-specific lipases as well as non-specific lipases (Li et al., 2025; Zheng, Wang, et al., 2025). In enzymatic glycerolysis reactions, Novozym 435 (non-specific), Lipozyme RM (1,3-specific), and Lipozyme TL^IM^ (1,3-specific) are all capable of catalyzing DAG synthesis, yet they exhibit marked differences in catalytic activity, product yield, and TAG conversion efficiency (Liu & Akoh, 2024). These differences arise from their distinct regioselectivity and active-site architectures. Lipozyme RM, an sn-1,3 preferential lipase from Rhizomucor miehei, primarily esterifies fatty acids at the sn-1,3 positions, whereas Novozym 435, a non-specific lipase from Candida antarctica, randomly esterifies all three positions of the glycerol backbone. This regioselectivity disparity directly influences the acyl-enzyme intermediate formation and the final TAG molecular species distribution. In contrast, the utilization of solvent-extracted oil from sesame cake remains relatively underexplored. Therefore, exploring the preparation of high-value DAG oil from sesame cake solvent-extracted oil not only helps diversify the feedstock sources for DAG oil but also offers a new pathway to enhance the utilization efficiency and added value of sesame cake resources, holding significant research significance and application prospects.

To address the research gap, this study employed solvent-extracted oil from pressed sesame cake as the substrate for DAG oil preparation via immobilized lipase-catalyzed glycerolysis. The research objectives were fourfold: (1) to screen suitable lipases and systematically optimize key process parameters (substrate molar ratio, enzyme loading, reaction temperature, and time) using single-factor and orthogonal experiments; (2) to verify DAG formation and structural alterations through FTIR; (3) to evaluate changes in fatty acid composition between the DAG product and the original sesame oil; and (4) to characterize the variations in chromaticity and physicochemical indices. This study aims to establish a high-value utilization pathway for sesame solvent-extracted oil, provide a novel and sustainable feedstock for functional diacylglycerol oils, and offer theoretical and technical support for the resource utilization of sesame processing by-products as well as the diversified development of the functional lipid industry.

## 2 Materials and methods

### 2.1 Materials

Sesame cake was provided from Sichuan Zhongxin Food Co., Ltd. (Chengdu, Sichuan, China). Lipases Novozym 435 (10,000 PLU/g), Lipozyme RM (275 IUN/g), and Lipozyme TL^IM^ (250 IUN/g) were obtained from Novozymes (China) Biotechnology Co., Ltd. (Tianjin, China). The diacylglycerol standard (purity >99%), glycerol, and anhydrous sodium sulfate (≥99%, CAS: 7757-82-6) were purchased from Shanghai Macklin Biochemical Co., Ltd. (Shanghai, China). n-Hexane (≥97%, CAS: 110-54-3) was supplied by Tianjin Kermel Chemical Reagent Co., Ltd. (Tianjin, China). Isopropanol (≥99.7%, CAS: 67-63-0), chromatographic grade acetone (≥99.5%, CAS: 67-64-1), sodium hydroxide (≥96.0%, CAS: 1310-73-2), phenolphthalein (≥98%, CAS: 77-09-8), glacial acetic acid (≥99.6%, CAS: 64-19-7), chloroform (≥99.8%, CAS: 67-66-3), and diethyl ether (≥99.5%, CAS: 60-29-7) were purchased from Sinopharm Chemical Reagent Co., Ltd. (Shanghai, China). Formic acid (≥99.5%, CAS: 64-18-6) and methanol (≥99.5%, CAS: 67-56-1) were obtained from Merck KGaA (Darmstadt, Germany).

### 2.2 Extraction of sesame oil and lipase-catalyzed preparation of DAG

Mechanically pressed sesame cake was ground and passed through an 80-mesh sieve to ensure uniform particle size. The resultant powder was dispersed in n-hexane at a solid-to-liquid ratio of 1:4 (g/mL) in a conical flask, and the mixture was incubated in a shaking water bath (Model HH-2, Guohua Instrument Co., Ltd., Jiangsu, China) at 50 °C for 2 h to facilitate solvent penetration and lipid dissolution. Following extraction, the mixture was filtered, and the supernatant was transferred to a round-bottom flask. The solvent was entirely removed using a rotary evaporator at 60 °C under reduced pressure (0.08–0.09 MPa) under reduced pressure, and the recovered sesame oil was collected for subsequent DAG synthesis.

DAG was prepared via the lipase-catalyzed glycerolysis of the extracted sesame oil. Briefly, sesame oil (10 g) and glycerol were mixed at a molar ratio of 1:3 (oil:glycerol), followed by the addition of Novozym 435 lipase at an enzyme loading of 8% (w/w, relative to the total substrate mass). Once the mixture reached the desired temperature, the flask was placed in a thermostatic shaking water bath at 80 °C and allowed to react for 10 h. Upon completion, the reaction mixture was centrifuged at 3700 rpm for 10 min, and the upper oil phase, serving as the DAG-rich product, was collected for further analysis.

### 2.3 Screening of lipozyme catalysts

To determine the optimal catalyst for the glycerolysis reaction, three commercial immobilized lipases – Lipozyme TL^IM^, Novozym 435, and Lipozyme RM - were evaluated based on preliminary experiments and relevant literature. The catalytic efficiency of each lipase was assessed using the mass fraction of DAG as the primary evaluation index. The screening was conducted under the following baseline conditions: a substrate molar ratio (sesame oil to glycerol) of 1:3, an enzyme loading of 8% (w/w, based on total substrate mass), a reaction temperature of 80 °C, and a reaction time of 10 h.

### 2.4 Single-factor experiments and orthogonal experimental design

Following the selection of the optimal biocatalyst, single-factor experiments were conducted to systematically investigate the effects of key reaction parameters on the DAG yield. The assessed variables included reaction temperature (50, 60, 70, 80, and 90 °C), reaction time (8, 9, 10, 11, and 12 h), substrate molar ratio of oil to glycerol (1:1, 1:2, 1:3, 1:4, and 1:5), and enzyme loading (4%, 6%, 8%, 10%, and 12%, w/w).

Based on the preliminary results of the single-factor experiments, an L_9_ (3^4^) orthogonal array design was employed to further optimize the enzymatic synthesis conditions and evaluate the relative importance of the primary variables. The three levels for each factor were selected to bracket the conditions that gave the maximum DAG yield in the single-factor tests; accordingly, levels that produced markedly lower yields were excluded from the orthogonal design. The substrate molar ratio of sesame oil to glycerol (Factor A), enzyme loading (Factor B), reaction time (Factor C), and reaction temperature (Factor D) were selected as the four independent variables, with the DAG content serving as the comprehensive evaluation index. Specifically, the experimental domain encompassed three predefined levels for each parameter: substrate molar ratios of 1:2, 1:3, and 1:4; enzyme loadings of 6%, 8%, and 10% (w/w); reaction times of 9, 10, and 11 h; and reaction temperatures of 70, 80, and 90 °C. This robust statistical approach allows for the systematic determination of the optimal reaction parameters required to maximize DAG accumulation.

### 2.5 Determination of DAG and TAG

The contents of DAG and triglycerides (TAG) were determined by high-performance liquid chromatography (HPLC) according to the method described by Aryee et al. (2011), with minor modifications. Chromatographic separation was performed on a Dionex UltiMate 3000 HPLC system (Thermo Fisher Scientific Co., Ltd., Waltham, MA, USA) equipped with a refractive index (RI) detector and a silica column (250 mm × 4.6 mm, 5 μm). The mobile phase consisted of a mixture of n-hexane, isopropanol, and formic acid at a volume ratio of 15:1:0.03 (v/v/v). The flow rate was maintained at 1.0 mL/min, and the column temperature was set at 30 °C.

For sample pretreatment, the lipid sample was dissolved directly in the aforementioned mobile phase to yield a final concentration of 2 mg/mL. The mixture was vigorously vortexed to ensure complete homogenization and subsequently filtered through a 0.22-μm nylon syringe filter into an autosampler vial. The total chromatographic run time was set to 30 min per injection. Target lipid classes, including DAG and TAG, were qualitatively identified by comparing their retention times with those of the corresponding commercial standards. The relative mass fractions of the components were quantitatively calculated using the peak area normalization method. Data acquisition and chromatogram processing were executed using Chromeleon 7.2 software (Thermo Fisher Scientific Inc.).

### 2.6 Determination of fatty acids profile

The fatty acid composition of the lipid samples was determined according to the Chinese National Standard GB 5009.168, 2016, utilizing a rapid cold transesterification method. Briefly, 100 mg of the oil sample was accurately weighed into a 10-mL conical flask and dissolved in 4 mL of isooctane, assisted by gentle heating in a 65 °C water bath until complete dissolution. Subsequently, 200 μL of 2% (w/v) methanolic potassium hydroxide was added. The mixture was vigorously vortexed for 1 min to convert the glyceride-bound fatty acids into their corresponding fatty acid methyl esters (FAMEs). Once the solution is clarified, 1.0 g of sodium bisulfate was added to neutralize the excess alkali. Following phase separation, the upper organic layer was filtered through a 0.45-μm organic syringe filter, and the filtrate was collected for gas chromatography (GC) analysis.

The GC analysis was executed on a Scion 456 GC system (Bruker Scientific Instruments, Inc., Hamburg, Germany) equipped with a flame ionization detector (FID), a Varian CP-8400 autosampler, and an Agilent J&W HP-88 capillary column (100 m × 0.25 mm, 0.20 μm film thickness; Agilent Technologies, USA). The temperatures of the injector and FID were maintained at 280 °C and 270 °C, respectively. The oven temperature program was initiated at 100 °C (held for 13 min), ramped to 180 °C at a rate of 10 °C/min (held for 6 min), further increased to 200 °C at 1 °C/min (held for 20 min), and finally raised to 230 °C at 4 °C/min (held for 10.5 min). Nitrogen was utilized as the carrier gas at a constant flow rate of 1.3 mL/min. The injection volume was 1.0 μL with a split ratio of 100:1. Individual fatty acids were identified by comparing their retention times with those of commercial FAME standard mixtures, and their relative mass fractions were quantified using the peak area normalization method. Data acquisition was performed using the Compass Chromatography Data System 3.0 (Techcomp Group).

### 2.7 Fourier transform infrared spectroscopy (FTIR)

The structural functional groups of the DAG and TAG samples were characterized using a Frontier FTIR spectrometer (PerkinElmer, Waltham, MA, USA) combined with the traditional KBr pellet technique. Briefly, 200 mg of spectroscopic-grade, dehydrated KBr powder was pressed into a transparent pellet at 10 MPa for 30–60 s. A thin liquid film was formed by applying a micro-drop of the oil sample onto the surface of the KBr pellet, which was subsequently placed under an infrared lamp at 80 °C to eliminate trace ambient moisture. The infrared spectra were recorded in the mid-IR region from 4000 to 400 cm^−1^ over 64 cumulative scans. A blank KBr pellet was measured prior to the samples to subtract the background spectrum. All acquisitions were conducted at a constant room temperature of 25 °C, and spectral visualization was performed using Origin 2024 software (OriginLab Corporation).

### 2.8 DAG and TAG color determination

The visual color attributes of the DAG and TAG samples were evaluated using a WN700D colorimeter (Weifu Optoelectronics Technology Co., Ltd., Shenzhen, China), following the methodology described by Gila et al. (2023). Prior to analysis, the instrument was thoroughly calibrated using standard black and white reference tiles. The liquid oil samples were carefully transferred into standard 10 mm × 10 mm × 30 mm quartz cuvettes to ensure a uniform optical path and flat reading surface. The measurement probe was placed flush against the optical window of the cuvette, and the color parameters were recorded. The results were expressed in the CIELAB color space, where *L*^⁎^ denotes lightness (0 = black, 100 = white), *a*^⁎^ represents the red-green axis (+ = red, − = green), and *b*^⁎^ indicates the yellow-blue axis (+ = yellow, − = blue).

### 2.9 Determination of physicochemical characteristics

The physicochemical properties of the DAG and TAG samples, including acid value (AV), iodine value (IV), *p*-anisidine value (*p*-AV), and peroxide value (POV), were determined in accordance with the respective Chinese National Standards. Specifically, the AV was determined via alkalimetric titration following the Chinese National Standard GB/T 5530-2005 and the results were expressed as mg KOH/g. The IV was measured following the Chinese National Standard GB/T 5532-2022, 2022 and the results were reported as g I₂/100 g. The *p*-AV was analyzed spectrophotometrically following the Chinese National Standard GB/T 24304-2024, 2024 and the results were reported as a dimensionless value. The POV was quantified via the iodometric method following the Chinese National Standard GB 5009.227-2023, 2023 and the results were uniformly expressed as g/100 g.

### 2.10 Statistical analyses

All experiments were performed in independent triplicates, and the analytical results are expressed as the means ± standard deviations (SD). Statistical analyses were performed using SPSS software (Version 27.0; IBM Corp., Armonk, NY, USA). For pairwise comparisons between the TAG and DAG samples, significant differences were assessed using an independent-samples *t*-test. For multiple group comparisons (e.g., single-factor experiments), data were subjected to one-way analysis of variance (ANOVA), followed by Duncan's multiple range test. Data visualization and graphical representations were executed using Origin Pro 2024 (OriginLab Corp., Northampton, MA, USA) and GraphPad Prism 10.1.2 (Dotmatics, Cambridge, UK). The value of *p* < 0.05 was considered statistically significant.

## 3 Results and discussion

### 3.1 Screening of lipases

**Fig. 1:**
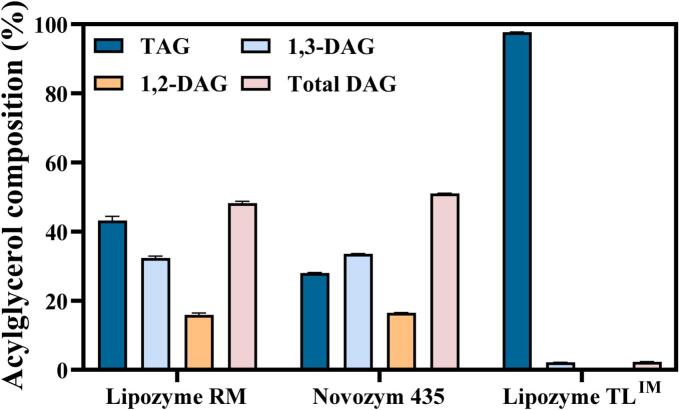
Effect of different lipases on the lipid composition during the enzymatic glycerolysis of sesame oil.

Under identical reaction conditions, the three commercial immobilized lipases exhibited drastically different catalytic performances in the glycerolysis of sesame oil (Fig. 1). When Lipozyme TL^IM^ was employed, the relative TAG content in the product remained exceedingly high at 97.68%, indicating that almost no substrate conversion occurred. In stark contrast, the TAG content in the Lipozyme RM and Novozym 435 groups plummeted significantly to 43.23% and 28.03%, respectively (*p* < 0.05), with Novozym 435 demonstrating the most robust TAG cleavage capability. The total DAG yields for the Novozym 435 and Lipozyme RM groups reached impressive levels of 51.06% and 48.25%, respectively, both significantly outperforming the Lipozyme TL^IM^ group (a mere 2.32%, *p* < 0.05). Regarding the distribution of isomers, the Novozym 435 group yielded the highest 1,3-DAG content (33.58%), slightly surpassing the Lipozyme RM group (32.38%). Concurrently, the 1,2-DAG levels in these two active groups were comparable (15.87% and 16.48%, respectively), both overwhelmingly exceeding those of the largely inactive Lipozyme TL^IM^ group (2.15% for 1,3-DAG and 0.17% for 1,2-DAG).

The dramatic variance in the catalytic activity of lipases is fundamentally governed by the physicochemical properties of their immobilization support (Adlercreutz, 2013). The notably low catalytic efficiency of Lipozyme TL^IM^ can be primarily attributed to its highly hydrophilic silica gel carrier (Kristensen et al., 2005). In a system with a relatively high concentration of glycerol, the highly polar glycerol molecules are prone to extensively adsorb onto the hydrophilic silica surface, forming a dense hydrophilic layer. This classic “glycerol coating effect” establishes a robust physical barrier that severely hinders the diffusion of highly hydrophobic TAG substrates into the enzyme's active center (Dossat et al., 1999). Yeoh et al. (2009) corroborated a similar phenomenon in the enzymatic glycerolysis of palm oil, observing that Lipozyme TL^IM^ possessed virtually no catalytic ability in the absence of auxiliary adsorbents. Additionally, the distinct microbial origins of Lipozyme RM (from Rhizomucor miehei) and Lipozyme TL^IM^ (from Thermomyces lanuginosus) may confer inherent differences in their catalytic behavior, as lipase properties including activity and selectivity are highly sensitive to the enzyme's structural features and can be greatly altered by immobilization strategy and reaction conditions (Fernandez-Lafuente, 2010). In stark contrast, Novozym 435 employs a hydrophobic macroporous acrylic resin as its support, whose hydrophobic microenvironment effectively repels the excessive accumulation of glycerol on the enzyme surface, thereby maintaining an unobstructed mass transfer channel at the oil-glycerol biphasic interface and ensuring efficient contact between the substrate and the enzyme (Ortiz et al., 2019). Comprehensively considering the TAG conversion rate, maximal DAG accumulation, and robust mass transfer tolerance in high-glycerol systems, Novozym 435 demonstrated unparalleled superiority and was definitively selected as the exclusive biocatalyst for subsequent optimizations.

### 3.2 Effects of processing parameters on TAG conversion and DAG isomer distribution

#### 3.2.1 Effect of substrate molar ratio on lipid distribution

**Fig. 2:**
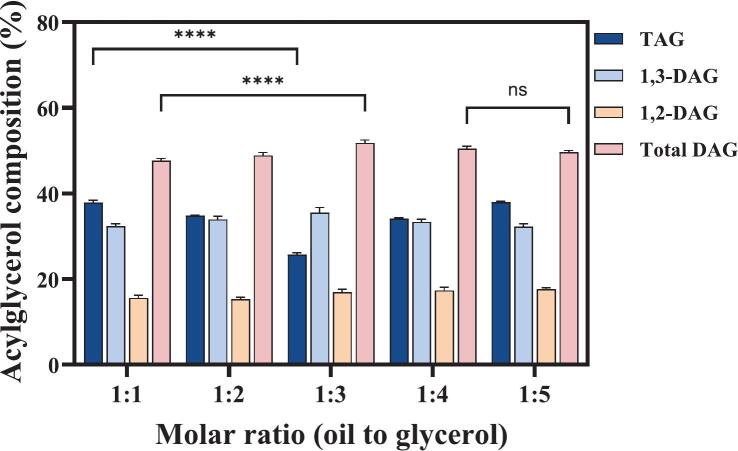
Effect of substrate molar ratio on the lipid composition during the enzymatic glycerolysis of sesame oil.

The consumption of TAG and the accumulation of total DAG exhibited opposite hyperbolic trends. As the molar ratio increased from 1:1 to 1:3, the relative TAG content decreased significantly from 37.90% to a minimum of 25.71%, concurrently driving the total DAG production to its peak (51.84%) (Fig. 2). However, further increasing the amount of glycerol to a ratio of 1:5 led to an unfavorable rebound in TAG content (38.00%) and a corresponding decline in DAG accumulation. Valério et al. (2010) also observed in the enzymatic glycerolysis of olive oil that as the glycerol-to-oil molar ratio increased from 3:1 to 6:1, the yields of monoacylglycerol (MAG) and DAG increased substantially, but further increases to 8:1 or 9:1 led to a decline in product yields. Regarding isomer distribution, the 1,3-DAG content peaked at 35.54% at a ratio of 1:3 before declining, while the 1,2-DAG level plateaued after reaching its maximum (16.96%) at the same ratio. Notably, the proportion of 1,3-DAG remained uniformly and significantly higher than that of 1,2-DAG across all investigated ratios (*p* < 0.05).

The observed phase of lipid conversion can be elucidated by the interplay between reaction equilibrium and interfacial mass transfer. At a lower glycerol ratio (1:1), the insufficient supply of hydroxyl donors restricted the forward thermodynamic drive, resulting in a high TAG residue. Elevating the ratio to 1:3 effectively pushed the equilibrium toward DAG synthesis according to Le Chatelier's principle. However, excessive glycerol (molar ratio ≥ 1:4) proved detrimental to DAG yield. This is primarily attributed to the high polarity of excessive glycerol, which tends to intensively adsorb onto and coat the surface of the immobilized enzyme support, creating a hydrophilic physical barrier (Criado & Otero, 2010). This heavily restricts the diffusion of the highly hydrophobic TAG into the enzyme's active pockets, escalating mass transfer resistance (Tran et al., 2016). Furthermore, abundant localized glycerol could trigger deep glycerolysis, converting the newly formed DAG into MAG and thus decreasing the final target yield. As reported by Roberta et al. (2010), a high glycerol-to-oil molar ratio of 3:1 favors MAG formation (up to 53 wt%), whereas lower ratios are more favorable for higher DAG production. The persistent predominance of 1,3-DAG over 1,2-DAG is largely attributed to acyl migration: despite the non-positional specificity of Novozym 435, the initially formed 1,2-DAG is sterically hindered and thermodynamically unstable, leading to its spontaneous intramolecular rearrangement into the more stable 1,3-DAG isomer during prolonged incubation (Mao et al., 2025; Sánchez et al., 2016). Considering both the maximal DAG efficiency and TAG consumption, a substrate molar ratio of 1:3 was selected for subsequent process optimization.

#### 3.2.2 Effect of reaction time on lipid distribution

**Fig. 3:**
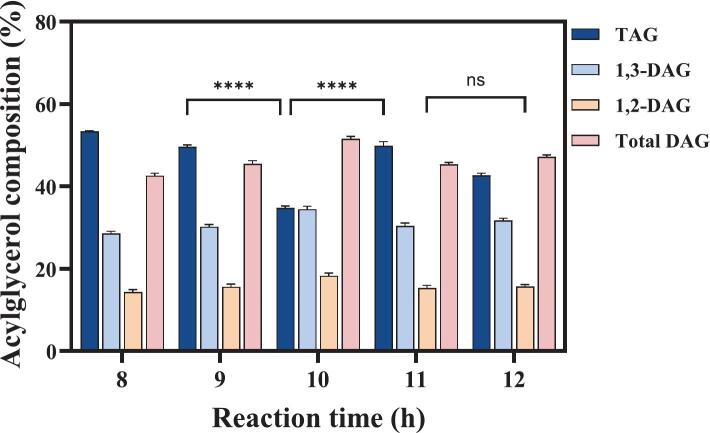
Effect of reaction time on the lipid composition during the enzymatic glycerolysis of sesame oil.

The lipid composition of the enzymatic glycerolysis system underwent a significant time-dependent evolution (Fig. 3). During the initial phase (8–10 h), the reaction was heavily dominated by the rapid cleavage of TAG, whose content plummeted from 53.35% at 8 h to a minimum of 34.79% at 10 h, before showing fluctuations upon further extended incubation. Conversely, the trajectory of total DAG displayed the classical accumulation pattern of an intermediate product, steadily increasing from 42.60% to a peak of 51.59% at 10 h, and subsequently declining to 47.14% at 12 h. Regarding isomer evolution, the trend of 1,3-DAG closely mirrored that of total DAG, reaching its zenith (34.50%) at 10 h. In contrast, the 1,2-DAG isomer increased from 14.37% to 18.33% during the first 10 h and then essentially plateaued within a narrow range of 15.33%–15.71% thereafter.

These dynamic lipid conversion trajectories are governed by the intrinsic kinetics of enzymatic glycerolysis, which operate as a typical consecutive reversible reaction. In the early driven stage (8–10 h), the rate of TAG consumption overwhelmingly exceeded the rate of DAG cleavage, resulting in a substantial accumulation of the DAG intermediate. However, prolonging the reaction beyond 10 h proved counterproductive. At this stage, the highly concentrated DAG became susceptible to deep glycerolysis, wherein it was further cleaved into MAG (Zou, Ai, Lee, et al., 2026). Furthermore, extended thermal exposure gradually drove the entire multiphase system toward an overarching thermodynamic equilibrium, leading to a slight drop in the target yield. The distinct kinetic behaviors of the DAG isomers are fundamentally intertwined with acyl migration (Mao et al., 2023). As precisely documented by Laszlo et al. (2008), the half-life for the isomerization of 1,2-DAG to 1,3-DAG is approximately 15.8 h at 80 °C. Therefore, extended reaction times provided a sufficient temporal window for the sterically hindered and energy-rich 1,2-DAG to spontaneously rearrange into the more thermodynamically favored 1,3-DAG. This mechanistic thermodynamic shift perfectly elucidates why 1,2-DAG generation stagnated in the later stages while 1,3-DAG consistently maintained its absolute dominance. Balancing the maximal DAG accumulation and overall reaction efficiency, 10 h was finalized as the optimal reaction duration.

#### 3.2.3 Effect of reaction temperature on lipid distribution

**Fig. 4:**
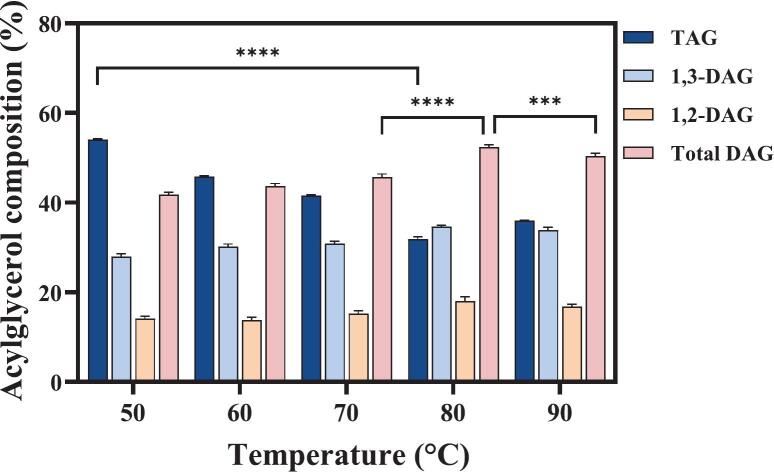
Effect of reaction temperature on the lipid composition during the enzymatic glycerolysis of sesame oil.

Reaction temperature exerted a profound impact on the lipid conversion process within the enzymatic glycerolysis system. As depicted in Fig. 4, the distribution of acylglycerol components shifted substantially across the evaluated temperature range of 50 °C to 90 °C. The relative content of TAG displayed a downward-then-upward trend, dropping continuously from 54.09% at 50 °C to a minimum of 31.85% at 80 °C. However, as the temperature further escalated to 90 °C, the TAG content rebounded slightly to 35.97%. Mirroring the TAG consumption trajectory, total DAG and its isomers demonstrated an inverse parabolic accumulation pattern. The total DAG level climbed steadily from 41.80% at 50 °C to a maximum of 52.44% at 80 °C, before slightly declining to 50.39% at 90 °C. Concurrently, both 1,3-DAG and 1,2-DAG reached their respective peak values (34.71% and 18.07%) at 80 °C and underwent a synchronous decrease at 90 °C.

The remarkable enhancement in DAG yield from 50 °C to 80 °C can be primarily attributed to the improved physicochemical properties of the multiphase system. Elevated thermal energy significantly reduced the macroscopic viscosity of the dense oil-glycerol mixture. This reduction drastically enhanced the interfacial mass transfer efficiency, facilitating the diffusion of highly hydrophobic TAG molecules into the active pockets of the immobilized lipase (Sun et al., 2009). Furthermore, increased molecular kinetic energy and collision frequency effectively drove the forward glycerolysis reaction. However, an excessively high temperature surpassed the optimal thermal tolerance threshold of Novozym 435 (Poojari & Clarson, 2013), likely inducing partial thermal denaturation of the enzyme's active conformation and thereby compromising its catalytic efficacy. Additionally, excessive thermal input might trigger reverse structural recombination once thermodynamic equilibrium is reached, collectively leading to the observed TAG rebound and the net decrease in DAG. Regarding isomer distribution, 1,3-DAG maintained its dominance across all temperature gradients, a phenomenon that became even more pronounced at higher temperatures. This decisively corroborates a thermodynamically driven mechanism in which high temperatures provided ample activation energy for the sterically hindered 1,2-DAG to overcome the energy barrier, thereby vastly accelerating its intramolecular acyl migration into the more energetically stable 1,3-DAG (Mao et al., 2023). Balancing the reaction efficiency and enzyme longevity, 80 °C was determined as the optimal temperature for DAG synthesis.

#### 3.2.4 Effect of enzyme loading on lipid distribution

**Fig. 5:**
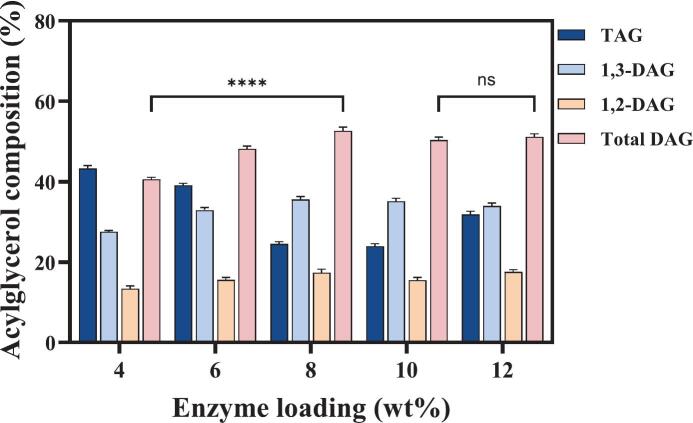
Effect of enzyme loading on the lipid composition during the enzymatic glycerolysis of sesame oil.

The concentration of the biocatalyst acts as a critical kinetic determinant governing the reaction rate and product distribution in the enzymatic glycerolysis system. As depicted in Fig. 5, the variation in enzyme loading significantly regulated the lipid conversion process across the evaluated range of 4% to 12%. When the enzyme dosage increased from 4% to 8%, the relative TAG content plummeted rapidly from 43.27% to 24.54%. Correspondingly, the accumulation of total DAG surged substantially from 40.65% to its zenith of 52.68%. Concurrently, the levels of both 1,3-DAG and 1,2-DAG peaked at this stage (35.62% and 17.40%, respectively). However, as the enzyme dosage further escalated to 10%, although TAG dropped marginally to its minimum (23.98%), the total DAG yield ceased to rise and declined to 50.36%. Notably, at a dosage of 12%, the total DAG yield fluctuated around 51.22%, while the TAG consumption stagnated and rebounded significantly to 31.81%. Regarding isomer distribution, 1,3-DAG consistently maintained a significant superiority over 1,2-DAG across all investigated enzyme gradients, with their relative proportion remaining dynamically stable after reaching the optimal enzyme loading.

This multiphasic progression vividly reflects the dynamic equilibrium between available catalytic sites and mass transfer efficiency in a multiphase enzymatic reaction. At lower enzyme dosages (4%–8%), the system was restricted by a finite number of active catalytic centers. Consequently, increasing the immobilized lipase concentration directly amplified the effective collision frequency between the substrates (TAG and glycerol) and the enzyme's active pockets, drastically accelerating the reaction cascade. Nevertheless, supersaturated enzyme loading (e.g., ≥ 10%) proved counterproductive. Excessive solid particle loading significantly increased the macroscopic viscosity of the system, leading to poor dispersion of the immobilized enzyme within the oil-glycerol multiphase mixture. This mixing limitation not only impeded effective mass transfer between substrates and enzymes but also created localized high enzyme concentrations that catalyzed the deep glycerolysis of newly formed DAG into MAG (Remonatto et al., 2015), thus decreasing the net target yield. Although a 10% enzyme loading resulted in a marginally higher TAG conversion than 8%, the highest total DAG yield was obtained at 8%. Balancing the maximization of target product accumulation and the necessity to minimize enzyme costs in industrial applications, 8% was designated as the optimal enzyme loading for this process.

### 3.3 Analysis of orthogonal experimental results

Based on the suitable parameter ranges established by the single-factor experiments, an L_9_ (3^4^) orthogonal array was employed to investigate the interactive effects among variables and to pinpoint the optimal parameter combination, utilizing the total DAG yield as the target metric (Table 1). Range analysis (R-value) was conducted to quantitatively evaluate the magnitude of each factor's influence on the DAG yield. As presented in Table 1, the R-values followed a descending order of D (4.8) > C (4.0) > B (3.9) > A (2.3). This hierarchy unambiguously indicates that reaction temperature (D) acts as the paramount parameter governing DAG generation, followed by reaction time (C) and enzyme loading (B), whereas the substrate molar ratio (A) exhibits the least sensitivity within the evaluated levels.

**Table 1:** L9 (3^4^) orthogonal array design and results for the optimization of DAG yield.

Test No.	A	B	C	D	Scheme	DAG yield (%)
1	1	1	1	1	A_1_B_1_C_1_D_1_	43.78
2	1	2	2	2	A_1_B_2_C_2_D_2_	49.90
3	1	3	3	3	A_1_B_3_C_3_D_3_	50.68
4	2	1	2	3	A_2_B_1_C_2_D_3_	52.15
5	2	2	3	1	A_2_B_2_C_3_D_1_	45.40
6	2	3	1	2	A_2_B_3_C_1_D_2_	51.04
7	3	1	3	2	A_3_B_1_C_3_D_2_	46.27
8	3	2	1	3	A_3_B_2_C_1_D_3_	52.95
9	3	3	2	1	A_3_B_3_C_2_D_1_	52.16
K_1_	144.36	142.20	147.76	141.34		
K_2_	148.59	148.25	154.21	147.21		
K_3_	151.38	153.87	142.35	155.78		
k_1_	48.12	47.40	49.25	47.11		
k_2_	49.53	49.42	51.40	49.07		
k_3_	50.46	51.29	47.45	51.93		
R-value	2.3	3.9	4.0	4.8		
Rank	D > C > B > A					
Optimal solution	A_3_B_3_C_2_D_3_					

As the primary determinant, reaction temperature (D) profoundly dictated the catalytic thermodynamics of Novozym 435 and the physical state of the multiphase system. Based on the evolution of k-values, as the temperature escalated from 70 °C (k₁ = 47.11) to 90 °C (k₃ = 51.93), the DAG yield exhibited a significant upward trajectory. This is primarily attributed to the decreased viscosity of glycerol at elevated temperatures, which significantly accelerates the reaction rate. The impact of reaction time (C) ranked second. Its k-value peaked at 10 h (k₂ = 51.40) before declining distinctly at 11 h (k₃ = 47.45). This trajectory corroborates the dynamic equilibrium nature of intermediate accumulation within the glycerolysis network: the optimal intermediate balance between TAG cleavage and DAG enrichment was achieved at 10 h. Conversely, excessively prolonged incubation invariably triggers deep hydrolysis or reverse acyl-transfer side reactions within the DAG pool, culminating in the unfavorable depletion of the target product.

Enzyme loading (B) also manifested a positive catalytic driving effect, with its k-value escalating stepwise as the dosage increased (from k₁ = 47.40 to k₃ = 51.29). Notably, although the substrate molar ratio (A) registered the lowest R-value, the highest glycerol proportion (level A3, 1: 4) still exhibited the maximum potential for DAG synthesis (k₃ = 50.46). This phenomenon unveils a profound synergistic mechanism within the multivariable system: in the orthogonal combinations, high temperature (90 °C) and elevated enzyme loading (10%) successfully neutralized the kinetic disadvantages, such as high viscosity and steric hindrance-typically induced by excess glycerol. Consequently, the thermodynamic potential of the redundant glycerol was fully liberated, utilizing its strong chemical potential to forcefully drive the reaction equilibrium toward DAG generation. By synthesizing the range analysis and the optimal trajectory of the k-values for each factor, the optimal parameter combination for the Novozym 435-catalyzed preparation of sesame oil-based DAG was determined to be A₃B₃C₂D₃. Specifically, this corresponds to a substrate molar ratio of 1:4, an enzyme loading of 10%, a reaction time of 10 h, and a reaction temperature of 90 °C. Under these synergistic conditions, the multiphase catalytic barriers can be maximally overcome, achieving the highly efficient and targeted enrichment of DAG from sesame oil.

### 3.4 Fatty acid composition analysis

**Fig. 6:**
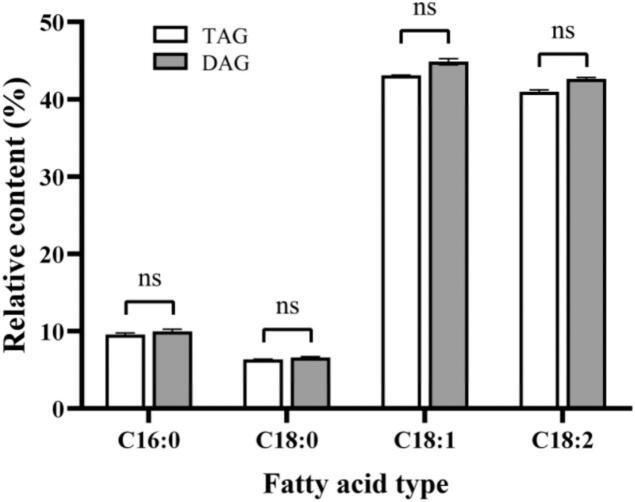
Comparison of fatty acid composition between sesame oil TAG and DAG.

Four principal fatty acids (FAs) were identified in the prepared sesame oil-based glyceride mixture, predominantly comprising palmitic acid (C16: 0), stearic acid (C18: 0), oleic acid (C18: 1), and linoleic acid (C18: 2) (Fig. 6). This FA profile is highly consistent with the characteristic native composition of typical sesame oil (Feng et al., 2025). Unsaturated fatty acids (UFAs) overwhelmingly dominated the system, with oleic and linoleic acids acting as the core constituents, jointly accounting for over 80% of the total FAs in the glycerides. This compositional characteristic not only signifies that the enzymatic glycerolysis product completely retains the exceptional nutritional value of sesame oil but also highlights the excellent mildness of the selected Novozym 435-catalyzed system despite the heating protocol.

During the conversion from TAG to DAG, the relative contents of all individual FAs exhibited no significant differences between the two lipid fractions (*p* > 0.05). Specifically, the relative contents of the core UFAs, C18: 1 and C18: 2, were maintained at 44.85% and 42.64% in the DAG fraction, which were essentially commensurate with those in the residual TAG fraction (43.11% and 40.98%, respectively). Similarly, the distribution of saturated FAs, such as C16:0 (TAG: 9.57%, DAG: 9.96%) and C18:0 (TAG: 6.35%, DAG: 6.60%), remained highly stable across both glyceride classes. This is likely because enzymatic glycerolysis is fundamentally a lipase-catalyzed transfer of acyl groups from the TAG backbone to free glycerol molecules, which primarily involves the specific hydrolysis and reconstitution of ester bonds without destroying the original carbon skeletons or the spatial isomeric structures of the fatty acids (Nicholson & Marangoni, 2021). Furthermore, given sufficient reaction time (10 h) and an appropriate temperature, the exchange of acyl groups among different glycerol backbones trended toward thermodynamic equilibrium. This ultimately resulted in a statistically random and uniform distribution of each FA across the residual TAG and the newly synthesized DAG pool. The absolute stability of highly unsaturated components such as C18: 1 and C18: 2 further indicates that the enzymatic system, devoid of exogenous strong oxidants, effectively circumvents side reactions such as the oxidative cleavage of products, thereby maintaining high oxidative stability. This molecular-level consistency convincingly confirms that the enzymatically regulated process successfully achieves the highly efficient enrichment of the target functional structural lipid (DAG) while flawlessly preserving the unique native lipid profile.

### 3.5 Infrared spectral analysis

**Fig. 7:**
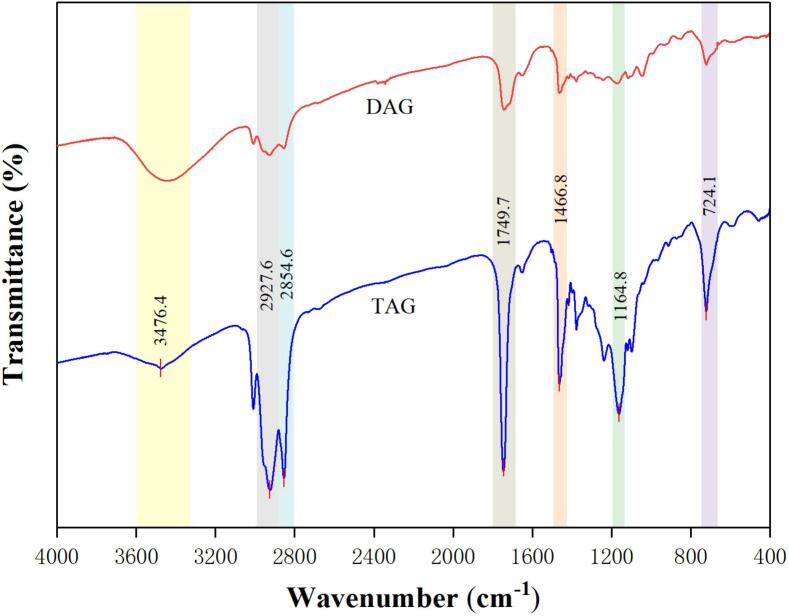
Comparison of FTIR spectra between sesame oil TAG and DAG.

The FTIR spectra (Fig. 7) demonstrated a high degree of spectral contour congruence between the initial sesame oil TAG and the enzymatic glycerolysis product DAG across the major characteristic absorption bands, indicating that the enzymatic process did not disrupt the primary molecular scaffold of the lipids. Specifically, both spectra exhibit intense asymmetric and symmetric stretching vibration peaks of methylene (—CH₂—) groups at 2927.6 cm^−1^ and 2854.6 cm^−1^, respectively. Coupled with the C—H bending vibration at 1466.8 cm^−1^ and the in-plane rocking vibration of long-chain methylenes at 724.1 cm^−1^, this confirms that TAG and DAG are highly homologous regarding their fatty acid carbon-chain architectures. This spectroscopic evidence mutually corroborates the aforementioned chromatographic analysis of the fatty acid composition.

Further comparison of the subtle variations in the polar functional group region reveals critical signatures at the molecular level that reflect their disparate physicochemical properties. The most prominent distinction is the emergence of a broad and distinct absorption band in the DAG spectrum within the range of 3200–3600 cm^−1^ (centered at approximately 3476.4 cm^−1^), which is definitively assigned to the stretching vibration of free hydroxyl groups (—OH). In contrast, the TAG spectrum displays a flat, non-absorbing baseline in this region, representing a typical characteristic of complete esterification. The appearance of the broad hydroxyl-related band suggests the generation of additional unesterified hydroxyl groups during glycerolysis. The introduction of free hydroxyl groups substantially transforms the physicochemical nature of lipid molecules. TAGs are predominantly hydrophobic, with negligible aqueous solubility. In contrast, DAGs are weak amphiphiles composed of a polar headgroup constituted by one free hydrophilic hydroxyl group and a nonpolar tail formed by two hydrophobic fatty acid chains. This structural duality imparts a permanent dipole moment to DAG molecules, significantly increasing their polarity compared with TAGs, and further endows them with distinct amphiphilic properties that enable unique interfacial behavior (Zou, Ai, Xie, et al., 2026). This enhanced amphiphilicity facilitates the adsorption of DAG molecules at oil-water interfaces, thereby improving emulsification performance and broadening their potential applications in functional food emulsions and encapsulation systems (Miklos et al., 2011). Concomitant with the generation of hydroxyl groups is the coordinated attenuation of ester-specific structural peaks. In the DAG spectrum, the absorption intensities of the ester carbonyl (C=O) stretching vibration at 1749.7 cm^−1^ and the C—O—C stretching vibration centered around 1164.8 cm^−1^ are notably lower than those in TAG, accompanied by broader and dampened peak contours. The synchronized weakening of these ester bond characteristic peaks confirms, at the molecular level, the absolute reduction in the number of ester groups (from three to two) during the conversion of TAG to DAG. Synthesizing these evolutionary patterns at the functional group level, the FTIR spectroscopy not only robustly validates the highly efficient conversion and enrichment of target DAG within the system but also unequivocally elucidates the intrinsic physicochemical divergence between diacylglycerols and triacylglycerols from the perspective of molecular bonding architecture.

### 3.6 Colorimetric analysis

**Fig. 8:**
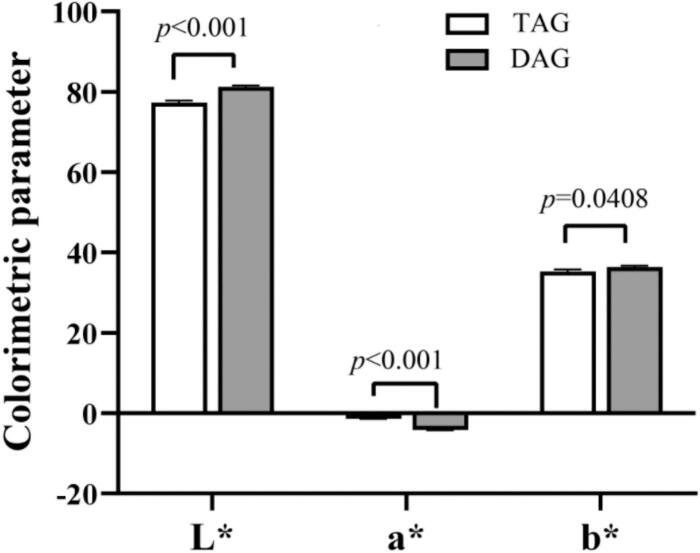
Comparison of colorimetric parameters and visual appearance between sesame oil TAG and DAG.

The evolution of colorimetric parameters provides an intuitive reflection of the comprehensive effects exerted by the enzymatic reaction and downstream processing on the physicochemical properties of the lipid system. As illustrated in Fig. 8, compared to the starting TAG, the lightness (*L*^∗^ value) of the resultant DAG sample increased highly significantly from 77.33 to 81.24 (*p* < 0.001), indicating a substantial improvement in the overall brightness and clarity of the product. This pronounced enhancement in lightness is intrinsically linked to alterations in the micro-components and physical state of the system. This significant enhancement in lightness further suggests that the glycerolysis reaction facilitated the extensive conversion of bulky TAGs into more polar DAGs and minor free fatty acids; this structural reconfiguration of the lipid matrix directly reduced the macroscopic refractive index of the system.

Regarding chromaticity indices, the two glycerides also exhibited distinctly divergent signatures (Fig. 8). The *a*^∗^ value of the DAG sample plummeted from −1.38 to −4.19 (*p* < 0.001), manifesting a conspicuous fading of redness and a deepening of greenness, whereas the *b*^∗^ value (representing the yellow-blue axis) displayed a statistically significant increase from 35.28 to 36.37 (*p* = 0.0408). This specific hue shift profoundly unravels the evolutionary trajectory of native pigments in sesame oil under the combined influence of thermal processing and a remodeled lipid microenvironment. During the prolonged isothermal enzymatic reaction, carotenoids, which dominantly dictate red-yellow hues, are highly susceptible to thermal oxidative degradation (Singh & Cama, 1974). The consequent massive depletion of red components serves as the primary driving force for the drastic negative shift in the *a*^∗^ value. Furthermore, the preferential degradation of red pigments disrupted the original pigment balance, allowing the underlying green tones to be visually exposed. Consequently, chlorophylls assumed a relatively dominant coloring role within the DAG system, ultimately bestowing the functional DAG product with a uniquely brighter, yellowish-green natural visual appearance.

### 3.7 Physicochemical properties analysis

**Fig. 9:**
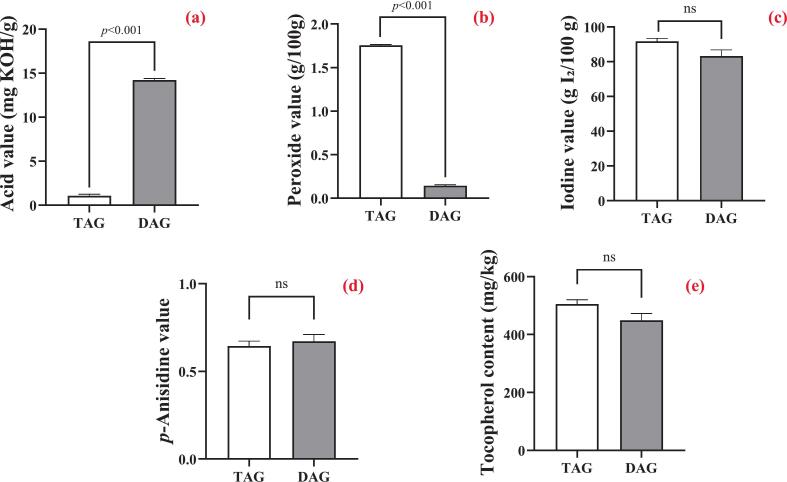
Comparison of fundamental physicochemical properties between sesame oil TAG and DAG: (a) Acid value, (b) Peroxide value, (c) Iodine value, (d) *p*-Anisidine value, (e) Tocopherol content.

Enzymatic glycerolysis not only drives the directed reconstruction of the glyceride backbone but is also inevitably accompanied by the dynamic evolution of the system's fundamental physicochemical properties. These macroscopic indices directly reflect the hydrolysis balance and oxidation state of the reaction system (Fig. 9). The AV is a crucial macroscopic indicator characterizing the enrichment degree of free fatty acids in lipid systems (Yu et al., 2012). As illustrated in Fig. 9(a), following the enzymatic reaction, the AV of the DAG sample surged dramatically from 1.01 mg KOH/g in the initial TAG to 14.14 mg KOH/g (*p* < 0.001). This marked increase is primarily attributed to the fact that, in a heated microenvironment containing excess polar glycerol and trace moisture, the lipase-catalyzed transesterification, while efficiently generating the target diacylglycerols, unavoidably induced profound hydrolytic side reactions, leading to extensive liberation of free fatty acids from the glycerol backbone (Azócar et al., 2011; Fregolente et al., 2008).

In stark contrast to the intensified hydrolysis, the levels of primary and secondary oxidation products in the system exhibited entirely divergent evolutionary trajectories before and after the reaction (Fig. 9). The results demonstrate that the POV of the DAG fraction plummeted from 1.75 g/100 g to 0.14 g/100 g (*p* < 0.001), whereas the *p*-AV, representing secondary oxidation products, showed no significant difference (increasing marginally from 0.6075 to 0.665; *p* = 0.3641). The massive dissipation of primary oxidation products (lipid hydroperoxides) is largely ascribed to their thermal instability and decomposition during prolonged heating and mechanical stirring. Concurrently, the unique bioactive lignans in sesame oil (such as sesamin) exerted a potent endogenous synergistic antioxidant effect within the system (Wan et al., 2015). More importantly, the relative stability of the *p*-AV indicates that although primary hydroperoxides dissociated, the relatively mild conditions of the enzymatic reaction effectively suppressed the deep cascade of lipid oxidation chain reactions. This prevented their massive cleavage into volatile short-chain aldehydes and ketones (undesirable secondary metabolites) (Grebenteuch et al., 2021), thereby fundamentally safeguarding the flavor and sensory quality of the DAG product.

Beyond the hydrolysis and oxidation states, the dynamic changes in the IV and endogenous antioxidant content further validated the preservation of core oil quality by this enzymatic process at the molecular level. IV primarily characterizes the average degree of unsaturation of the lipid system; the IV of the DAG sample (83.33) exhibited no significant alteration compared to the raw TAG (92.38) (*p* = 0.1070). This aligns perfectly with the aforementioned microstructural analyses of fatty acid composition and FTIR, confirming that the enzymatic catalysis did not compromise the spatial configuration and absolute abundance of polyenic double bonds. Meanwhile, γ-tocopherol, an exceptionally vital natural antioxidant in sesame oil, decreased slightly from 550 mg/kg to 450 mg/kg post-reaction, though the difference did not reach statistical significance (*p* = 0.1411). This non-significant minor depletion likely originated from a shift in the phase partition coefficient of γ-tocopherol caused by the massive presence of polar solvent (glycerol) in the system, coupled with minor non-specific physical adsorption by the macroporous resin carrier of the immobilized lipase. Overall, the comprehensive evaluation of various physicochemical indices demonstrates that enzymatic glycerolysis under moderate conditions achieves the highly efficient recombination of structural lipids while maximally preserving the fragile unsaturated carbon chain backbones and native lipophilic micronutrients of the sesame oil system.

## 4 Conclusions

The present study demonstrated that Novozym 435-catalyzed enzymatic glycerolysis serves as an effective structural modification strategy for sesame oil. Systematic single-factor and orthogonal evaluations revealed that the precise coordination of key processing parameters successfully overcame the mass transfer resistance inherent in the highly polar multivariable system, circumventing multiphase kinetic barriers to achieve the targeted conversion of DAG. Compared to conventional high-temperature chemical processing, this relatively mild enzymatic pathway exhibited distinct advantages in quality preservation. It flawlessly maintained the integrity of polyunsaturated carbon chain backbones and maximally retained endogenous lipophilic micronutrients. Furthermore, the massive generation of polar DAG components, coupled with the preferential thermal degradation of specific pigments, endowed the product with a uniquely brighter, yellowish-green appearance. Concurrently, the potent endogenous synergistic antioxidant network of the sesame oil system successfully suppressed the deep cascade of lipid oxidation, fundamentally safeguarding the oxidative stability and nutritional quality of the final product. Nevertheless, while this enzymatic reconfiguration successfully bestowed unique amphiphilic properties upon the glyceride backbone, it inevitably induced profound hydrolytic side reactions, leading to a notable elevation in the AV of the system.

To address this specific limitation and bridge the gap to industrial formulation, future research should prioritize: (1) mechanistic investigations into the accumulation kinetics of free fatty acids during multiphase glycerolysis, alongside the development of targeted green deacidification or hydrolytic suppression strategies; and (2) systematic evaluations of the interfacial behavior and nutritional delivery potential of this antioxidant-rich sesame oil-based DAG within functional food emulsions or baking matrices, thereby fully actualizing its commercial applicability.

## CRediT authorship contribution statement

**Li Zhou:** Writing – original draft, Methodology, Funding acquisition, Data curation. **Yue Mu:** Writing – original draft, Methodology. **Jin Zhang:** Methodology, Investigation, Data curation. **Meiyu Zheng:** Writing – original draft, Methodology, Investigation. **Lei Chen:** Visualization, Methodology. **Qingyang Zhang:** Investigation. **Jinglun Zhou:** Writing – original draft. **Zhengting Zhu:** Methodology. **Shu Wang:** Data curation. **Bin Li:** Methodology, Investigation. **Zhigang Hu:** Resources. **Kangyu Zhao:** Resources, Conceptualization. **Dongping He:** Resources, Methodology. **Fenfen Lei:** Supervision, Project administration, Funding acquisition.

## Declaration of competing interest

The authors declare that they have no known competing financial interests or personal relationships that could have appeared to influence the work reported in this paper.

## Data Availability

The datasets generated for this study are available on request to the corresponding author.
